# A MICRO CAREGIVING INITIATION: LATE-LIFE REMARRIAGE OF OLDER WIDOWED ARAB MUSLIM MEN IN ISRAEL

**DOI:** 10.1093/geroni/igad104.0955

**Published:** 2023-12-21

**Authors:** Chaya Koren, Hanan Ali Morshed

**Affiliations:** University of Haifa, Haifa, HaMerkaz, Israel; University of Haifa, Haifa, HaZafon, Israel

## Abstract

New caregiving solutions are increasingly in demand within families undergoing rapid modernization processes such as the Arab Muslim family in Israel. According to patriarchal collectivist values, caregiving for aging men is provided by women within the extended Arab Muslim family. Modernization within the Arab Muslim society has enabled women to acquire higher education and increased employment opportunities outside the household. Therefore, older Arab Muslim widowers in need for caregiving can depend less on their daughters or daughter’s in-law and as such seek other solutions. The aim of this presentation is to describe and examine the caregiving role of late life remarriage for older Arab Muslim widowers. Using a phenomenology framework, semi-structured qualitative interviews for understanding the experience of late life remarriage and its meaning were conducted with 14 Arab Muslim widowers who remarried between age 70 and 80, to never married middle-aged women. Findings describe and illustrate caregiving as a primary motivation for late life remarriage, reasons for caregiving as motivation to remarry and the wife’s role as located between caregiver and spouse. Conclusions discuss the meaning of late life remarriage as a micro caregiving solution, dealing with modernization processes alongside preserving patriarchal collectivist values and gender-based roles.

